# Chronology of COVID-19 Cases on the Diamond Princess Cruise Ship and Ethical Considerations: A Report From Japan

**DOI:** 10.1017/dmp.2020.50

**Published:** 2020-03-24

**Authors:** Eisuke Nakazawa, Hiroyasu Ino, Akira Akabayashi

**Affiliations:** Department of Biomedical Ethics, School of Public Health, Faculty of Medicine, The University of Tokyo, Tokyo, Japan; Tokyo Metropolitan Geriatrics Hospital, Tokyo, Japan; Division of Medical Ethics, Department of Population Health, New York University School of Medicine

**Keywords:** COVID-19, Diamond Princess cruise ship, public health ethics, Japan

## Abstract

The Diamond Princess cruise ship has been anchored at the Yokohama port in Japan since February 3, 2020. A total of 691 cases of the coronavirus disease 2019 (COVID-19) infection had been confirmed as of February 23. The government initially assumed that the infection was not spreading aboard and therefore indicated that any persons who either tested negative for the virus or were asymptomatic should immediately disembark. However, on February 5, the government set a 14-day health observation period because of the severity of the infection. Passengers confirmed to be free from infection began disembarking on Day 15 (February 19) of the quarantine. The effectiveness and validity of infection control, justification for the timing of inspections, and even the nature of COVID-19 itself now are all in question. The ethical considerations related to cruise ship infection control include the reasonable justification for isolation, the psychological fragility and quality of life of the isolated passengers and crew members, the procedural justice inherent in a forced quarantine, and the optimization of control measures.

The international coordination framework and the global ramifications of such outbreaks should be reevaluated by the international community. Denying a ship’s entry based on local politics is incompatible with global justice. Events such as these require an international response and global regulations that seek to reduce disparities.

## FOURTEEN-DAY HISTORY OF THE COVID-19 EPIDEMIC ON THE DIAMOND PRINCESS

### Background

**FIGURE 1:**
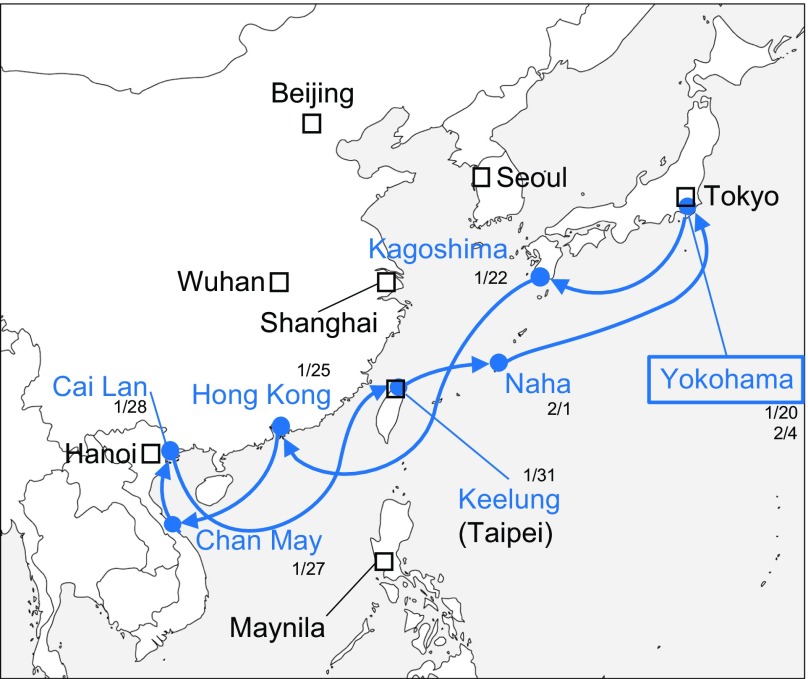
Itinerary of the Diamond Princess Cruise Ship From January 20 to February 4, and the Geopolitical Map.^3^

The Diamond Princess cruise ship (cruise number M003) has been anchored at the Yokohama Port since February 3, 2020. Aboard the Diamond Princess were 2666 passengers, 1281 of whom were Japanese and 1045 crew members from a combined total of 56 countries.^1,2^ The ship departed from Yokohama Port, Japan, on January 20, 2020, and proceeded to Hong Kong on January 25; Chan May Port, Vietnam, on January 27; Cai Lan, Vietnam, on January 28; Keelung, Taiwan, on January 31; and Naha, Japan, on February 1. It was scheduled to return to its departure point in Yokohama on February 4 to complete its 16-day voyage^3^ (Figure 1).

On February 1, 2020, Hong Kong’s government announced that pneumonia due to the coronavirus disease 2019 (COVID-19) was confirmed in Mr A, an 80-year-old male passenger^*^ on the Diamond Princess who had disembarked on January 25.^4^ Later, on February 3, the Diamond Princess docked off Daikoku Pier at Yokohama Port. No immediate word was issued on when the ship would be permitted to dock.^5^

### Changes in the Number of COVID-19-Infected Patients on the Diamond Princess and Actions of the Japanese Government

**FIGURE 2:**
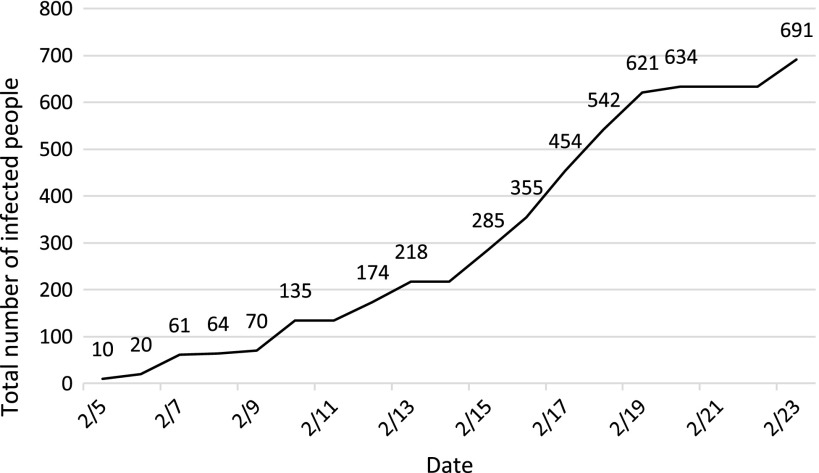
Infected Passengers and Crew Members on the Diamond Princess. (All Data Were Retrieved From Press Releases By the Ministry of Health, Labour and Welfare of Japan.^6^)

The number of COVID-19-infected patients on the Diamond Princess is shown in Figure 2.^6^ The first 10 cases were confirmed on February 5, and, by February 23, when passengers began disembarking, the number of confirmed cases had risen to 691. Infection had also been confirmed in 5 quarantined officers and health care workers on February 21, in addition to 2 deaths on February 20^7^ and another on February 23.^8^

A chronological summary of the responses of the Japanese government is shown in Table 1. Various policies and decisions had been made by the Japanese government. A quarantine of the Diamond Princess (based on Quarantine Law) was ordered at Okinawa Port on February 1, leading to the issuing of a provisional quarantine certificate.^9^ Based on this, entry was permitted for all passengers and crew members under the Immigration Control and Refugee Recognition Act.^10^ However, later that day, the results of Mr A’s test for COVID-19 were released by the Hong Kong government. When the Diamond Princess arrived at Yokohama Port on the night of February 3, the government resumed the quarantine of the passengers and crew members under the Quarantine Law.^11^ On February 5, 10 people tested positive for the virus, and quarantine was commenced under the Quarantine Law (Day 1 of the quarantine). COVID-19 testing was initially limited to those with symptoms such as fever, but quickly expanded to high-risk individuals – aged passengers and those with a chronic illness. On Day 5 of the quarantine (February 9), the Japanese government started considering the possibility of conducting testing on all passengers and crew members at the end of the 14-day health observation period. With these considerations, the policy was changed so that a virus test would be conducted on all passengers on Day 11 of the quarantine (February 15). However, this policy did not mention crew members.

**TABLE 1:** Chronological Summary of the Japanese Government’s Responses

Date	Quarantine Days	Virus Testing Policy	Immigration Restrictions and Disembarkation
February 1, 2020			The Diamond Princess stops at Okinawa and has completed quarantine upon entering Japan.^11^
February 3			The ship arrives at Yokohama. The quarantine in Okinawa has been canceled and passengers and crew members have been re-quarantined on the ship.^11^
February 5	Day 1	Screening for the virus is implemented in the aged or people with chronic disease, even if asymptomatic.^1^	A 14-day health observation period is set for all passengers and crew members; disembarkation is limited.^1,50^
February 9	Day 5	Japanese government begins considering the possibility of testing all passengers and crew members at the end of the 14-day health observation period.^38,51^	
February 13	Day 9		A policy allows the aged to disembark, specifically, older people over the age of 80 years who are in cabins without windows and suffer from chronic illness.^17,38^
February 15	Day 11	Policy regarding the inspection of all passengers is implemented.^52,53^	
February 19	Day 15		Disembarkation begins of passengers confirmed as not being infected.^54^
February 21	Day 17		Disembarkation of 970 asymptomatic passengers with negative test results is completed. Passengers should avoid outings, unless urgent, for 2 weeks and measure body temperature daily. Their health status should be checked regularly at health centers.^55^
February 22	Day 18	Crew members will be tested for viruses in the future.^55^	Approximately 1300 people, including crew members, foreigners waiting for charter aircraft, and passengers who were in close contact with or in the same room as a person who tested positive, are aboard.^50^ Disembarkation of crew members is undecided.^18^

The government initially assumed that the infection was not spreading aboard and therefore indicated that any persons who either tested negative for the virus or were asymptomatic should immediately disembark.^12^ Several newspapers reported optimistically that passengers would be able to disembark on February 4.^13,14^ However, after 10 tests came back positive for the virus, the Japanese government and Ministry of Health, Labour and Welfare (MHLW) became concerned.^12,15^ They decided that they could not rule out the possibility of community transmission by disembarked passengers or crew members during the incubation period.^16^ Because of the severity of the infection, a 14-day health observation period was established, and entry restrictions were enforced on the passengers and crew members. On Day 9 of the quarantine, a policy was adopted to allow the aged to disembark.^17^ Passengers who were confirmed to be free from infection began disembarking on Day 15 of the quarantine, and this disembarkation was completed on Day 17 of the quarantine (February 21). However, 1300 passengers remained aboard, and the details regarding the disembarkation of crew members were yet to be determined.^18^

### Reactions of Countries Around the World

On February 15 (11 days after isolation), the US Embassy in Japan announced that the US government was arranging charter aircraft to evacuate its citizens off the Diamond Princess. Chartered passengers would be quarantined for 14 days after arriving in the United States.^19^ In accordance with this policy, 2 US government charter planes with US citizens departed for the United States on the morning of February 17 (13 days after isolation).^20^ Thereafter, many countries, including Canada, Hong Kong, Australia, and South Korea, announced policies to evacuate their citizens.^21–24^

On February 21, the Australian government announced that 2 of its citizens returning to Australia on a chartered aircraft were infected with COVID-19.^25^ The Israeli government also confirmed COVID-19 infection in 1 female Israeli passenger.^26^

### Denied Entry of Another Cruise Ship: The MS Westerdam

On February 6, Prime Minister Shinzo Abe revealed that Japan would refuse entry to foreigners on the cruise ship MS Westerdam, which was scheduled to dock in Okinawa on February 8, unless there were special reasons to do otherwise.^27^ The MS Westerdam departed from Hong Kong on February 1 and traveled through Kaohsiung on February 5 on its way to Ishigaki Port in Japan. Because of suspected COVID-19 pneumonia, the Japanese government prohibited the MS Westerdam from docking at Naha Port.^28^ The ship was refused entry under the Immigration Control Law, Article 5.1 (14), entitled “Persons with a reason to be deemed likely to harm Japan’s interests and public interest.” This clause, which has been applied only once since 1945,^9^ was cited for the reason for the response after a National Security Council meeting that positioned COVID-19 as a national security issue.^29^ As a result, the MS Westerdam canceled its itinerary and was placed in a situation where it was unable to decide where to go.^30^

On February 12, Cambodia agreed to let the MS Westerdam dock and allow passengers to disembark.^31^ On February 13, the MS Westerdam entered Sihanoukville, Cambodia. Prime Minister Hun Sen told the media, “The real disease is fear, not the virus.”^32^ World Health Organization (WHO) Director-General Tedros Adhanom praised Cambodia’s actions as indicative of “international solidarity” insituations where cruise ships are denied entry “without an evidence-based risk assessment.”^33^

On February 15, the Malaysian government announced that it had detected COVID-19 in an 83-year-old American woman who had disembarked from the MS Westerdam and entered Malaysia.^34^

## FACTS TO BE EXAMINED

### Validity of Infection Control

Japanese infectious disease control is currently critically discussed around the world. “The response from Japan is chaotic and ad hoc,” criticized Russia’s Foreign Ministry spokeswoman Maria Zakharova on February 10.^35^ An officer of the US Centers for Disease Control and Prevention responded in media interviews that they were concerned about the high risk to the health of passengers.^36^

With the understanding that it is extremely difficult to prevent infection reliably in a cruise ship environment, Japan’s infectious disease control has been criticized for being lax. Dr Michael Ryan of the WHO stated, “Sometimes there are environments in which disease can spread in a more efficient way,” and pointed out that cruise ships, in particular, are known to accelerate spread occasionally.^37^ Nathalie MacDermott of King’s College London noted, “Obviously the quarantine hasn’t worked, and this ship has now become a source of infection.”^37^

From a public health perspective, it is too early to judge whether Japan’s infection control was appropriate, and this must be assessed in detail in the future. We must examine how government policies, collaboration with international organizations, recommendations from infectious disease experts, and media coverage influenced the health of passengers and crew members on the Diamond Princess. Given that public health and the legal system are closely linked, the interrelationship between laws and regulations, such as the Infectious Diseases Law, the Quarantine Law, and the Immigration Control Law, as well as their relationships with international law, should be considered. In addition, the ship’s flag state doctrine and emergency ship rescue protocols need to be analyzed. The doctrine is a principle of international law that does not apply to domestic law or provide administrative authority to foreign ships. Following this principle, emergency ship rescue is associated with numerous legal difficulties.

### Timing of Inspections

It was suggested earlier that a comprehensive virus test should be conducted immediately on all passengers and crew members on the Diamond Princess. However, when dealing with insufficient medical resources, the selection of appropriate test subjects can lead to emergency medical resource allocation issues.

At first, the MHLW limited inspections to those who showed symptoms, such as fever, and had been in close contact with passengers confirmed as being infected. Later, the MHLW began to consider screening all passengers and crew members.^38^ The challenge was to ensure the ability to inspect 3000 people in a short period of time. It was reported that a high-ranking official of the MHLW had expressed doubts about the feasibility of this plan, specifically stating, “Testing by private companies will cost a lot. Prefectural and municipal public health institutes have little experience in testing for this new coronavirus. I’m not sure if it is possible to test such a large number of people.”^38^ Similarly, Yoshihide Suga (Chief Cabinet Secretary) stated, “As things stand now, it will be really tough to test (all remaining passengers).”^38^

Determining how macro-level measures can be taken to address the temporary scarcity of medical resources in an emergency is a challenging public policy issue. To ensure patient safety and timely medical intervention, further careful consideration must also be given to the appropriate timing to undertake inspections for all passengers and crew members.

### Nature of COVID-19

What kind of virus is COVID-19? What is the appropriate infection control? Public health policy at the time of the outbreak must offer a reasonable strategy based on available epidemiological data and knowledge of disease characteristics. Decision-makers must be aware of and sensitive to the plethora of unconfirmed data, misinformation, and media hype that tend to incite public fear and lead to political policies that end up doing more public harm than good. Estimating the infectivity and severity of COVID-19 directly affects the basis of infection control; however, the true value of its infectivity and severity will only be known retrospectively. Therefore, we now have no choice but to resort to collective intelligence – the understanding of an infectious disease and its control must be based on an academic consensus involving as many infectious disease control specialists and public health experts as possible, accompanied by appropriate legal and ethical guidance.

Since the outbreak on the Diamond Princess occurred in a very closed environment, the cases should be examined to obtain infectious disease data, which can be used for analysis, such as identifying the route of COVID-19 transmission and infectivity. The diversity of the Diamond Princess passengers and crew in terms of race and ethnicity can provide valuable data to help governments prepare for the global spread of COVID-19 in the future.

## PUBLIC HEALTH ETHICS OF THE DIAMOND PRINCESS

### Isolation and Justice

Isolating a cruise ship carrying passengers infected with COVID-19 is conditionally consistent with the principle of justice. The dilemma between isolation and human rights is a classic and fundamental issue in public health ethics, and, thus, it is difficult to propose concrete arguments that solve the dilemma completely. Hence, from the perspective of justice, the necessary conditions for acceptable isolation should be examined.

The quality of life (QOL) of the passengers and crew members on an isolated ship must at least exceed the minimum to live a reasonable life. To do so, the individuals must be provided with the basic medical care needed to stay healthy. The quarantined Diamond Princess had already reported a shortage of medicines on Day 2 of the quarantine (February 5).^39^ The MHLW responded quickly and supplied the medications needed by patients with diabetes and heart disease by Day 7 of the quarantine (February 10); however, there was still a shortage.^38^ In addition, passengers and crew members on isolated vessels are subject to severe stress; therefore, mental support must also be provided.

American bioethicist Arthur Caplan said, “Boats are notorious places for being incubators for viruses. It’s only morally justified to keep people on the boat if there are no other options.”^37^ WHO executive Michael Ryan said, “We need to balance the health and welfare of the people on that ship from many nationalities against the obvious need to prevent any further spread (of the virus) within the Japanese community.”^40^ Isolating a cruise ship carrying passengers infected with COVID-19 is only allowed in consideration of this balance.

### Psychological Condition and Quality of Life of Isolated Passengers and Crew Members

Ingenuity must be constantly pursued to enhance the QOL of those isolated on a cruise ship. QOL depends on an individual’s mental state, as well as material goods. Above all, we want to focus on mental state. Junior high school and high school students in Hokkaido sent a video message to the passengers and crew members on the Diamond Princess saying (in English), “We are with you!”^41^ A banner titled, “Yokohama Stand by You! [sic]” was displayed on 3 pleasure boats around the wharf where the Diamond Princess was moored. Eleven days after isolation (February 14), when fatigue reached its peak, SoftBank and LINE (an IT company) cooperated at the request of the MHLW to provide 2000 iPhones for passengers to send drug requests and receive health consultations free of charge.^42^ Such goodwill gifts enrich our minds. The underlying value is to empathize with the isolated passengers and crew members, that is, to experience the same feeling of suffering.

Mental Health First Aid is important in responding to the acute mental stress caused by catastrophic events. Although a disaster psychiatric assistance team (DPAT) was dispatched, concerns were raised about measures to control infectious diseases aboard, and ongoing support of mental health was at stake.^43^ The disruption of ongoing support can be stressful, so careful consideration is needed when introducing a DPAT.

### Procedural Justice

Procedural justice is the ethical minimum in choosing an option that cannot always be expected to have positive results in difficult situations. The ideal of public health activities in emergencies meets the greatest happiness principle: maximum happiness for the maximum number of people. Procedural justice complements this utilitarian judgment. In this case, procedural justice is also the expression of the virtues of policy-makers, such as justice and honesty. Emergency public health activities must involve the disclosure of information and the elimination of opacity.

## THE DIAMOND PRINCESS IN TERMS OF PUBLIC HEALTH PREPAREDNESS: THE NEED FOR A GLOBAL PERSPECTIVE

### Passenger–Crew Member and North–South Divides

The environment was different for passengers and crew members. The Diamond Princess has over 1000 crew members,^44^ most of whom are from low- and middle-income countries in Southeast Asia.^45^ Whereas passengers who require care may have access to private rooms, crew members do not^46^ and must continue to provide service to passengers who may be potentially infected. Crew members have the sole responsibility to provide general services to passengers and facilitate ship navigation, and are not trained medical professionals. Despite the difficulty of adopting medical professionalism,^47^ they are required to act like voluntary medical personnel.^48^ This is a matter related to the global social and economic structure.

### Is Refusal to Allow a Ship to Dock Justified?

Globally, the decision to allow cruise ships with suspected infected patients to dock should be considered from the viewpoints of geopolitics and the availability of medical resources. Geopolitical considerations include the proximity of the cruise ship to infected areas and secure routes for transporting patients suspected of being infected to health care facilities.

In addition, the capacity to provide health care should be considered. In Japan, the reported number of influenza cases has been remarkably low throughout the 2019–2020 flu season, with less than half of the cases reported than in previous years.^49^ This is likely due to the increasing public awareness of hygiene associated with the COVID-19 pandemic. The Japanese health care system was therefore well positioned to provide care for passengers and crew members on cruise ships.

Global justice seeks the rectification of unfair burdens among global regions. Denying a cruise ship entry to a port because of a COVID-19 outbreak poses various ethical issues. The acceptance criteria for patients suspected of being infected should be based on (1) the nation’s geopolitical status and (2) the nation’s ability to provide adequate health care. Disembarking passengers and crew members should be offered care on land, and all steps to prevent the spread of the infection should be taken.

### From Local Politics to a Global Perspective: International Collaboration for Public Health Preparedness

The COVID-19 pandemic is a global challenge. Sticking to dominant and local politics makes global issues less visible. The lack of a global perspective results in unhappy people who are irrationally disadvantaged between nations. Cruise ships traveling across oceans with passengers and crew members of various nationalities in a closed environment are essentially international. The international society must consider the COVID-19 pandemic as a universal issue and address difficulties through mutual collaboration. This requires transparent procedures, the assurance of accountability, rapid and flexible international cooperation, a spirit of charity, and a theory of justice that seeks to correct misfortunes and disparities.

## CONCLUSION

The COVID-19 pandemic not only threatens the health of the populations of infected countries, but also has a significant social and economic impact. In the first section, we focused on the outbreak that occurred aboard the Diamond Princess and described the history of ongoing infections by reviewing publicly accessible newspaper reports and government public relations. In addition, we presented issues that must be scrutinized from the perspectives of public health (in the *Facts to Be Examined* section) and public health ethics. Finally, in terms of public health preparedness in the future, we highlighted the need for international collaboration and global justice.

Because information on the biological nature and epidemiological characteristics of COVID-19 is complex, ethical requirements include optimizing isolation conditions, giving consideration to the QOL to those who are isolated, and ensuring the transparency of information disclosure. Considering that a cruise ship is a miniature international society, international coordination and global justice are needed to reduce social and economic disparities.
